# Screening of Ethanol, Petroleum Ether and Chloroform Extracts of Medicinal Plants, *Lawsonia inermis* L. and *Mimosa pudica* L. for Antibacterial Activity

**DOI:** 10.4103/0250-474X.70492

**Published:** 2010

**Authors:** A. Akter, F. A. Neela, M. S. I. Khan, M. S. Islam, M. F. Alam

**Affiliations:** Biotechnology and Microbiology Laboratory, Department of Botany, University of Rajshahi, Rajshahi - 6205, Bangladesh

**Keywords:** Medicinal plants, antibacterial activity, organic extract, bacteria

## Abstract

Organic extracts (ethanol, petroleum ether and chloroform) of two medicinal plants *Lawsonia inermis* L. and *Mimosa pudica* L. were proven for antibacterial properties against 15 Gram-positive and Gram-negative human pathogenic bacteria. Among the three types of extracts tested, ethanol extract was found to possess maximum antibacterial activity. The diameter of the zone of inhibition of bacterial growth showed that Gram-negative bacteria are more sensitive than Gram-positive bacteria to plant extracts. Between the two plants species studied, *Lawsonia inermis* extract showed more antibacterial activity compared to *Mimosa pudica* extract.

Medicinal plants have played a significant role in ancient traditional systems of medication in many countries. They are rich source of bioactive compounds and thus serve as important raw materials for drug production[1,2]. Now-a-days multiple drug resistance has developed due to the indiscriminate use of commercial antimicrobial drugs commonly used in the treatment of infectious disease[3]. This situation forced scientists to search for new antimicrobial substances. Therefore, there is a need to develop alternative antimicrobial drugs for the treatment of infectious diseases from medicinal plants.

*Lawsonia inermis* L. and *Mimosa pudica* L. belong to the family of Lythraceae and Fabaceae, respectively. Both plant species are well-known for their medicinal properties and commonly distributed in Bangladesh. *L. inermis* is traditionally used for the treatment of headaches, migraine, albinism, skin abrasions and ulcers, burns, smallpox, leprosy boils, wounds, some mycotic infections. It was also used for the treatment of scalp and hair infections and ailments[4]. It has antibacterial[5], anticancer[6] and antituberculostatic activity[7,8]. *M. pudica* has been used traditionally in the treatment of various ailments including alopecia, diarrhoea, dysentery, insomnia, tumour and various urogenital infections[9]. It has also wound healing[10], antibacterial[11,12] and antioxidant activity[11]. The purpose of this study was to screen of these two medicinal plants extracts that could be useful for the development of new tools as antibacterial agents for the control of both Gram-positive and Gram-negative human pathogenic bacteria.

The young leaf and stem (twig) of *L. inermis* L. and *M. pudica* L. were collected from Rajshahi University Flora, Bangladesh and identified at the Department of Botany, Rajshahi University, Rajshahi-6205. These specimens are preserved in the herbarium, Department of Botany, Rajshahi University, Rajshahi-6205, Bangladesh (*Lawsonia inermis* L.- Voucher Specimen No. 36 and *Mimosa pudica* L.- Voucher Specimen No. 52).

Following Gram-positive *Staphylococcus aureus* (BMLRU1002), *Bacillus cereus* (BMLRU1004), *Staphylococcus haemolytica* (BMLRU1006), *Bacillus subtilis* (BMLRU1008), *Bacillus megaterium* (BMLRU1010), *Sarcina lutea* (BMLRU1012) and Gram-negative *Escherichia coli*-B (BMLRU1001), *Klebsiella* sp. (BMLRU1003), *Klebsiella pneumoniae* (BMLRU1005), *Pseudomonas aeruginosa* (BMLRU1007), *Salmonella typhi* (BMLRU1009), *Shigella dysenteriae* (BMLRU1011), *Shigella shinga* (BMLRU1013), *Shigella sonnei* (BMLRU1015) and *Pseudomonas* sp. (BMLRU1017) species were tested. The strains were collected from the International Center for Diarrhoeal Disease Research of Bangladesh (ICDDRB), Mahakhali, Dhaka, Bangladesh.

Cleaned fresh twig samples of *Lawsonia* and *Mimosa* were cut into small pieces, oven dried at 40°. Well pulverized 5 g powder of the plant sample was continuously shaken with 20 ml each of ethanol, petroleum ether and chloroform on a water bath shaker for 10 h. The extracts were filtered through the Whatman No.1 filter paper, and the solvents were evaporated using a rotary evaporator to obtain brownish dark sticky residues. The evaporated extracts were dissolved in the respective solvents (10 mg/ml).

Bacterial strains were grown on Luria-Bertani (LB) medium which was prepared using the following compositions: 10 g of yeast extract supplemented with 10 g of bacto peptone and 5 g of NaCl dissolved in 1 liter of water[2]. The pH was adjusted at 7.0 before solidifying with bacto agar (20 g/l). The medium was autoclaved at 15 lb/inch^2^ in pressures at the temperature of 121° for 20 min to ensure sterilization.

Disc diffusion method[13] was followed to test of the antimicrobial activity of *L. inermis* and *M. pudica* against the chosen 15 bacterial strains. Discs of 6 mm in diameter[14] were soaked with 10 μl of each extract (10 mg/ml) and air dried under aseptic condition inside the laminar flow and placed on seeded LB agar plates and incubated at 37° for 24 h. A 100 μl of bacterial suspension (108 cfu/ml) was used for spreading on LB agar plates. Only respective solvents (without extract) containing sterile blank discs were used as a negative control and tetracycline (30 μg/ml) was used as a positive control[13]. After incubation, the antibacterial activity of *L. inermis* and *M. pudica* was determined by measuring zone of inhibition in millimeter scale against individual studied bacteria. Each assay was carried out in triplicate.

Two-folds serial dilution method[15] was followed for MIC determination of studied plant extracts. Ten milligram of the semisolid extracts were dissolved in 2 ml of the respective solvent to get a concentration 5 mg/ml (5000 μg/ml) which was serially diluted to achieve 2500, 1250, 625, 312.5 and 156.25 μg/ml, respectively. One hundred microlitters of each concentration of test samples were added into the test tubes containing 9 ml bacterial suspension (10^8^cfu/ml), separately. Control test tubes contained only test organisms with distilled water instead of plant extract. The test tubes were incubated at 37° for 24 h. The least concentration of the plant extracts with no visible growth was taken as the MIC.

The results of the antibacterial screening are represented in Tables 1 and 2. Variable antibacterial effects of plant extracts was observed against bacterial strains and compared with the reference standard tetracycline. In ethanol extract of *L. inermis*, the zone of inhibition was ranging from 7.20 mm (*E. coli*) to 17.25 mm (*S. dysenteriae*). Lowest (156.25 μg/ml) and highest (2500 μg/ml) MIC was observed for *S. dysenteriae* and *E. coli*, respectively. In comparing with positive control it is shown that ethanol extract of *L. inermis* has good antibacterial properties. In petroleum ether extract highest and lowest zone of inhibition was 15.03 mm and 7.40 mm against *S. dysenteriae* and *S. lutea*, respectively and their corresponding MIC was 2500 μg/ml and 156.25 μg/ml. Here no zone of inhibition was observed for *B. cereus* and *E. coli*. In chloroform extract out of fifteen cases only in five cases, zones of inhibition were observed. This data is in close agreement with that published by other researchers[5,16,17]. Comparing among the three types of extracts, ethanol extract of *L. inermis* was found most effective for antibacterial activity especially on Gram negative bacteria.

**TABLE 1:** ANTIBACTERIAL ACTIVITY OF EXTRACTS OF *LAWSONIA INERMIS*

Bacterial species	Zone of inhibition (mm)/Minimum inhibitory concentration (μg/ml)						
	Ethanol extract	Petroleum ether extract	Chloroform extract	Negative control			Positive control
				Et	Pet	Chl	
Gram Positive							
*Staphylococcus aureus*	17.1±0.14 165.25	8.20 ±0.14 1250	8.30±0.14 1250	+	+	+	17.50±0.70
*Bacillus cereus*	11.1±0.13 625	+ <2500	+ <2500	+	+	+	20.21±0.14
*Streptococcus haemolytica*	9.20±0.12 1250	9.20±0.21 1250	+ <2500	+	+	25.01±0.41
*Bacillus subtilis*	12.2±0.07 625	10.12±0.14 625	+ <2500	+	+	+	24.50±0.36
*Bacillus megaterium*	10.35±.21 625	12.40±0.20 625	12.23±0.14 625	+	++	14.50±0.86
*Sarcina lutea*	15.4±0.14 156.25	7.40±0.14 2500	+ <2500	+	++	19.50±0.70
*Gram negative*							
*Shigella sonnei*	9.50±0.14 1250	8.50±0.14 1250	+ <2500	+	+	+	24.20±0.14
*Escherichia coli*	7.20±0.15 2500	+ <2500	+ <2500	+	+	+	18.33±0.14
*Shigella shinga*	9.25±0.07 1250	10.25 ±0.7 625	10.40±0.07 625	+	+	+	14.50±0.70
*Klebsiella pneumoniae*	9.10±0.14 1250	9.10±0.14 625	15.13±0.14 312.5	+	+	+	15.02±0.14
*Salmonella typhi*	12.4±0.28 625	10.35±0.21 625	+ <2500	+	+	+	23.50±0.12
*Klebsiella sp.*	15.15±0.7 625	12.15±0.07 312.5	+ <2500	+	+	+	14.50±0.14
*Pseudomonas sp.*	11.15±.14 625	9.04±0.07 625	+ <2500	+	+	+	22.25±0.35
*Pseudomonas aeruginosa*	8.20±0.28 1250	9.35±0.80 625	9.40±0.02 625	+	+	+	20.20±0.20
*Shigella dysenteriae*	17.25±.07 156.25	15.03±0.03 156.25	+ <2500	+	+	+	19.33±0.14

Values are represented as mean±SD of triplicate experiments; +, Bacterial growth; Et, ethanol; Pet, petroleum ether; Chl, chloroform.

**TABLE 2:** ANTIBACTERIAL ACTIVITY OF EXTRACTS OF *MIMOSA PUDICA*

Bacterial species	Zone of inhibition (mm)/Minimum inhibitory concentration (μg/ml)							
	Ethanol extract	Petroleum ether extract	Chloroform extract	Negative control			Positive control
				Et	Pet	Chl	
Gram positive							
*Staphylococcus aureus*	11.2+0.14 625	8.20±0.07 2500	+ >2500	+	+	+	16.16±0.14
*Bacillus cereus*	9.35 ±0.21 625	+ >2500	12.2 ±0.14 625	+	+	+	18.33±0.23
*Streptococcus haemolytica*	+ >2500	+ >2500	+ >2500	+	+	+	23.16±0.14
*Bacillus subtilis*	10.45±0.07 625	15.30±0.14 156.25	12.40±0.07 165.25	+	+	+	21.50±0.28
*Bacillus megaterium*	12.35±0.21 312.5	+ >2500	9.23±0.14 625	+	+	+	15.16±0.16
*Sarcina lutea*	+ >2500	+ >2500	+ >2500	+	+	+	18.30±0.33
Gram negative							
*Shigella sonnei*	+ >2500	+ >2500	+ >2500	+	+	+	23.20±0.14
*Escherichia coli*	+ >2500	+ >2500	+ >2500	+	+	+	19.50±0.28
*Shigella shinga*	10.25±0.7 1250	+ >2500	10.40±0.07 625	+	+	+	14.50±0.22
*Klebsiella pneumoniae*	9.50±0.23 312.5	+ >2500	8.15±0.23 2500	+	+	+	14.50±0.18
*Salmonella typhi*	10.4±0.14 625	10.25±0.07 625	+ >2500	+	+	+	22.50±0.12
*Klebsiella sp.*	+ >2500	+ >2500	+ >2500	+	+	+	17.03±0.22
*Pseudomonas sp.*	+ >2500	+ >2500	+ >2500	+	+	+	17.50±0.28
*Pseudomonas aeruginosa*	13.40±0.14 312.5	14.35±0.21 312.5	+ >2500	+	+	+	21.20±0.20
*Shigella dysenteriae*	13.20±0.14 312.5	+ >2500	+ >2500	+	+	+	20.50±0.28

Values are represented as meanμSD of triplicate experiments;+, Bacterial growth; Et, ethanol; Pet, petroleum ether; Chl, chloroform.

In ethanol extract of *M. pudica*, the highest and lowest zone of inhibition was 13.40 mm (MIC, 312.5 μg/ml) and 9.35 mm (MIC, 625 μg/ml) against *P. aeruginosa* and *B. cereus*, respectively. This data is in close agreement with Balakrishnan *et al*[12]. On the other hand, for petroleum ether and chloroform extract the corresponding highest zone of inhibition was observed 15.30 and 12.40 mm in *B. subtilis* with MIC value 156.25 μg/ml. Similar observations were made with *L. inermis*, where ethanol extract was found to exhibit most effective antibacterial activity against Gram-negative organisms. Negative control exhibited no zone of inhibition as expected whereas, positive control (disc containing antibiotics) exhibited zones of inhibition against all strains studied. Between the two plant species *L. inermis* showed more antibacterial activity than *M. pudica*.

The screening of plant extracts, has led to the discovery of many clinically useful drugs that now play major roles in the treatment of human diseases[18]. In the present work, the twig extracts of *L. inermis* and *M. pudica* have been determined for their antibacterial properties for controlling some Gram-positive and Gram-negative bacteria. Among the three extracts, ethanol extract was more effective than petroleum ether and chloroform extracts against Gram-negative bacteria than Gram-positive bacteria. Parekh and Chanda[19] also reported that alcoholic extract of 50 Indian plants is better than aqueous extract. Among them *Terminalia chebula, Mangifera indica* and *Eucalyptus citriodora* showed maximum antibacterial activity. Khan *et al*.[20] also reported that the ethanol extract of *Achyranthus aspera* was much affective in Gram-negative bacteria than Gram-positive one. These observations may be attributed to the nature of biological active components whose activity can be increased in the presence of ethanol. Several types of alkaloids, glycosides, steroids and proteins have been reported to have the antibacterial activity[21].

In conclusion, the results of the study showed the antibacterial activity of twig extracts of *L. inermis* and *M. pudica*. The antibacterial activity of the plant may be attributed to the various phytochemical constituents present in the crude extract. The work carried out was a basic approach to find out antibacterial activity residing in medicinal plants. This study indicated that medicinal plants could be a potential source of newer antibacterial agents. Further works on the types of phytoconstituents and purification of individual groups of bioactive compounds can reveal the exact potential of the plant to inhibit several pathogenic microbes and effective applications to control pathogenic bacteria causing severe illness in humans.
